# Decreased BDNF Release in Cortical Neurons of a Knock-in Mouse Model of Huntington’s Disease

**DOI:** 10.1038/s41598-018-34883-w

**Published:** 2018-11-19

**Authors:** Chenglong Yu, Chun Hei Li, Sidong Chen, Hanna Yoo, Xianan Qin, Hyokeun Park

**Affiliations:** 1 Division of Life Science, The Hong Kong University of Science and Technology, Clear Water Bay, Kowloon, Hong Kong, China; 2 Department of Physics, The Hong Kong University of Science and Technology, Clear Water Bay, Kowloon, Hong Kong, China; 3 State Key Laboratory of Molecular Neuroscience, The Hong Kong University of Science and Technology, Clear Water Bay, Kowloon, Hong Kong, China

## Abstract

Huntington’s disease (HD) is a dominantly inherited neurodegenerative disease caused by an increase in CAG repeats in the Huntingtin gene (*HTT*). The striatum is one of the most vulnerable brain regions in HD, and altered delivery of BDNF to the striatum is believed to underlie this high vulnerability. However, the delivery of BDNF to the striatum in HD remains poorly understood. Here, we used real-time imaging to visualize release of BDNF from cortical neurons cultured alone or co-cultured with striatal neurons. BDNF release was significantly decreased in the cortical neurons of zQ175 mice (a knock-in model of HD), and total internal reflection fluorescence microscopy revealed several release patterns of single BDNF-containing vesicles, with distinct kinetics and prevalence, in co-cultured cortical HD neurons. Notably, a smaller proportion of single BDNF-containing vesicles underwent full release in HD neurons than in wild-type neurons. This decreased release of BDNF in cortical neurons might lead to decreased BDNF levels in the striatum because the striatum receives BDNF mainly from the cortex. In addition, we observed a decrease in the total travel length and speed of BDNF-containing vesicles in HD neurons, indicating altered transport of these vesicles in HD. Our findings suggest a potential mechanism for the vulnerability of striatal neurons in HD and offer new insights into the pathogenic mechanisms underlying the degeneration of neurons in HD.

## Introduction

Huntington’s disease (HD), a dominantly inherited neurodegenerative disease, is caused by an expansion of CAG repeats in the Huntingtin gene (*HTT*)^1,2^. This CAG-repeat expansion in the gene results in an increased polyglutamine stretch in the translated mutant huntingtin protein, which plays a detrimental role in neurons^3–5^. Patients with HD exhibit severe involuntary motor dysfunction, psychiatric disturbances, and cognitive impairment caused by atrophy in several brain regions, among which the caudate nucleus and the putamen in the dorsal striatum are particularly vulnerable^6–8^. Several mechanisms have been proposed to explain the neuronal loss in the striatum in HD, including decreased levels of brain-derived neurotrophic factor (BDNF) in the striatum^6,9^.

BDNF is a small secreted protein (~14 kDa) that belongs to the neurotrophin family and is abundantly expressed in the adult brain^10–12^. Whereas pro-BDNF attenuates synaptic plasticity and exacerbates neuronal damage by activating the p75 neurotrophin receptor (p75NTR)^13,14^, mature BDNF plays an essential role in promoting neuron survival and synaptic activity^15,16^; BDNF also mediates the growth and maturation of neurons^17,18^. Because little BDNF mRNA is detected in striatal neurons^19–21^, these neurons are believed to receive BDNF primarily from the cortex^19,22^. BDNF levels in the striatum are markedly reduced in the conditional knockout mouse Emx-BDNF^KO^, in which BDNF expression in the cortex is abolished^22^. Conversely, disrupting BDNF transport from the cortex using colchicine causes BDNF to accumulate in the soma of cortical afferent neurons^19^.

The role of BDNF in HD pathogenesis has been extensively investigated, as BDNF plays a crucial role in neuron survival. Early studies using HD mouse models found that BDNF levels were diminished in both cortical and striatal neurons^23,24^, which agrees with the findings of studies regarding the striatum in HD patients^25^. Furthermore, insufficient BDNF production in cortical neurons was found to be associated with decreased striatal BDNF levels in YAC72 mice^23^, and impaired cortical transport was also reported to contribute to the reduction in BDNF levels in the striatum in HD mice^10,15^. However, the detailed mechanism of BDNF delivery during HD pathogenesis remains poorly understood. Here, we found that BDNF release was substantially decreased in cultured neurons from the zQ175 mouse, a knock-in model of HD that is relevant to human HD. We observed that single BDNF-containing vesicles have distinct release patterns and that the proportion of vesicles that undergo full release is altered in cortical projections close to striatal neurons in zQ175 mice. We also found altered basal-level transport of BDNF-containing vesicles in HD neurons from zQ175 mice. Finally, we found that applying BDNF to pure cultures of HD striatal neurons from zQ175 mice prevents their decreased soma size, which was observed in untreated pure HD striatal neurons in comparison with wild type neurons. Our results suggest that altered release and transport of BDNF-containing vesicles contributes to the impaired delivery of BDNF to the striatum in HD, thereby increasing the vulnerability of these neurons.

## Methods

### Animals

zQ175 knock-in mice were purchased from Jackson Laboratories and maintained in the Animal and Plant Care Facility at the Hong Kong University of Science and Technology (HKUST). Mice were housed with *ad libitum* access to food and water. Heterozygous mice were used for breeding. All experimental procedures were performed in accordance with the regulations of the Animal Ethics Committee at HKUST, and were approved by the Department of Health, Government of Hong Kong.

### Primary culture

Cortical and striatal neurons were cultured from heterozygous zQ175 pups and their wild-type (WT) littermates at postnatal day 0 (P0) after genotyping. Dissociated neurons were plated onto 12-mm coverslips (Deckglaser, Germany) and placed either in separate wells in 24-well plates or in adjacent compartments of culture dishes containing inserts (ibidi, Martinsried, Germany) for co-culture^26^. Coverslips were precoated with poly-D-lysine (Sigma). For co-cultures, the inserts were removed at 1 day *in vitro* (DIV 1), and the cultures were washed carefully with Neurobasal medium (Lifetech). Neurons were maintained in Neurobasal medium supplemented with 5% FBS (Hyclone), 2% B27 (Lifetech), and 0.5 mM Glutamax (Lifetech). To inhibit glial cell proliferation, 20 μM 5-fluoro-2′-deoxyuridine (Sigma) was applied at DIV 3. Neurons were maintained in a 5% CO_2_ incubator at 37 °C and used at DIV 14–21^27^.

### Calcium phosphate transfection and electroporation

Calcium phosphate transfections were performed in cultured cortical neurons at DIV 10 to express BDNF-pHluorin or BDNF-EGFP. Neurons were then returned to their original plates and used for experiments after DIV 13. The BDNF-pHluorin construct was kindly provided by Prof. Muming Poo (Institutes of Neuroscience, CAS, Shanghai). For co-cultures, cortical or striatal neurons were electroporated using the constructs for BDNF-pHluorin or pCMV (MinDis). iGluSnFR immediately before plating on ibidi dishes. The pCMV (MinDis). iGluSnFR was a gift from Prof. Loren Looger (Addgene plasmid #41732)^28^.

### Time-lapse imaging of BDNF-pHluorin release

**Figure 1:**
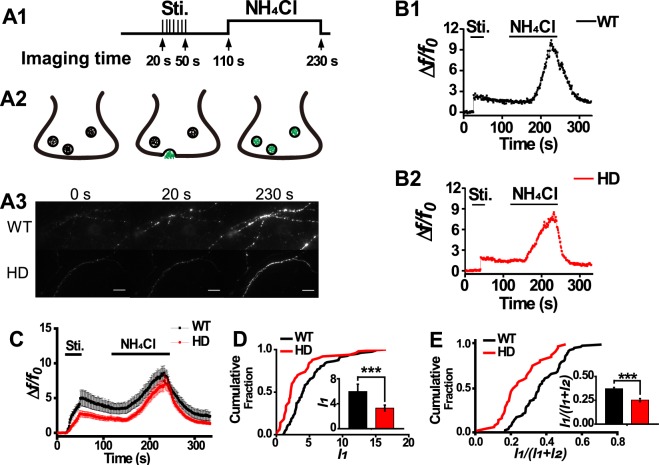
Release of BDNF-pHluorin in cortical neurons. (**A**) Schematic diagrams of the experiment paradigm and representative images. (A1) Schematic of the experimental paradigm. The 20 s before stimulation served as the baseline, which was followed by 10-Hz stimulation for 30 s; after a brief rest period, the neurons were perfused with NH_4_Cl for 120 s, and the bath solution was changed back to normal ACSF after 230 s. Schematic of neuronal response (A2) and representative images (A3) of WT and HD neurons at baseline (0 s), during stimulation (30 s), and during NH_4_Cl perfusion (230 s). (**B**) Representative relative fluorescence intensity traces of the BDNF-pHluorin in WT (B1) and HD (B2) cortical neurons. (**C**) Averaged relative intensity traces of BDNF-pHluorin obtained from WT and HD neurons. (**D**) Cumulative plot of peak intensity *(I*_*1*_) induced by stimulation. Inset, bar graph of the peak intensity in WT neurons (n = 81 puncta) and HD neurons (n = 50 puncta); the peak intensity in HD neurons was significantly lower compared with WT neurons (^***^p < 0.001, Mann-Whitney *U* test). (**E**) Cumulative plot of peak intensity ratio, calculated as [*I*_1_*/(I*_1_ + *I*_2_)]; *I*_1_ and *I*_2_ represent the peak intensities induced by stimulation and NH_4_Cl perfusion, respectively. Inset, bar graph of the peak intensity ratio in WT neurons and HD neurons; the peak intensity ratio in HD neurons was significantly lower compared with WT neurons (^***^p < 0.001, Mann-Whitney *U* test). Scale bar: 10 μm.

The microscopy setup and stimulation apparatus were built as previously described^29,30^. Time-lapse imaging at 1 Hz (exposure time 100 ms) was performed using an EMCCD camera (Andor iXon Ultra 897). Coverslips were mounted together with the imaging chamber (Warner, 64-0284 PH1 heated platform) onto an Olympus inverted microscope (Olympus IX73). Neurons were continuously perfused with normal artificial cerebrospinal fluid solution (ACSF) containing 120 mM NaCl, 4 mM KCl, 2 mM CaCl_2_, 2 mM MgCl_2_, 10 mM D-glucose, and 10 mM HEPES (pH 7.2–7.4, 300–310 mOsm/L). The bath solution was maintained at 37 °C using a heater (Warner, TC-344C), and the bath solution was changed to ACSF containing 50 mM NH_4_Cl for determining the total expression level of BDNF-pHluorin^27,31^. A 100× oil-immersion objective (Olympus) was used with a ZT488 rdc dichroic mirror and an ET 525/50 m emission filter for the GFP channel. A 488-nm and 532-nm laser (CrystaLaser) was used to image BDNF-pHluorin and FM4-64, respectively. Field stimuli (1-ms duration) were applied at 10 Hz using a Grass isolator (SD9, Grass Technologies) and custom-made parallel platinum wires. Stimulation, beam shutter, and the EMCCD camera were synchronized using pClamp 10.5 (Molecular Devices) and controlled using Andor SOLIS software (Andor). Neurons were incubated with 10 μM FM4-64 (Thermo Fisher Scientific) for labeling synaptic regions via spontaneous exocytosis and endocytosis^32,33^. FM4-64 was imaged using a ZT532 rdc dichroic mirror and an ET 605/70 m emission filter immediately before the time-lapse imaging of BDNF-pHluorin. Theta-burst stimulation (TBS), consisting of 10 trains of stimuli with a 5-s interval, with each train comprising 10 pulses at 5 Hz with 4 spikes at 100 Hz, was applied as previously reported^27^. The fluorescence intensities in the regions of interest (ROIs) were compared with the local background of two adjacent ROIs; the ROIs were regarded as synaptic regions if the FM4-64 signals were higher than the average intensity plus two standard deviations (*σ*) of the local background^29,34^. Field stimulation (300 stimuli at 10 Hz) was applied to trigger BDNF release in WT and HD cortical neurons. After stimulation, neurons were perfused with NH_4_Cl for 120 s and then the bath solution was changed back to normal ACSF at the end of imaging (Fig. 1A). Local expression of BDNF-pHluorin was identified by the peak intensity during NH_4_Cl perfusion. Images of WT and HD neurons were acquired alternately.

### Real-time imaging of exocytosis of single BDNF-containing vesicles

Total internal reflection fluorescence microscopy (TIRFM) was used to identify the release of single BDNF-containing vesicles. Live images were captured at 10 Hz with a 100-ms exposure time. To trigger single-vesicle release, a brief stimulation at 50 Hz for 6 s was used after imaging the baseline for 5 s. ACSF containing 0.6 μM bafilomycin A1 (EMD Millipore) was used to measure the contribution of re-acidification to the fluorescence changes in BDNF-pHluorin^27,35^. The bath solution was changed to MES buffer (normal ACSF with HEPES replaced with MES, and pH lowered to 5.5) to test whether the fluorescence decay was affected by vesicular movement from local positions^27,35^.

### Real-time imaging of BDNF-EGFP transport

Cortical neurons were transfected with BDNF-EGFP at DIV 10 using calcium phosphate. Transfected neurons were imaged after DIV 13. Real-time imaging was performed using the frame-transfer mode with an exposure time of 100 ms. Field stimuli were applied at 10 Hz for 60 s. Transport of BDNF-EGFP was analyzed using the ImageJ macro Kymolyzer^36^ both without and during electrical stimulation. The total travel length and the average speed were compared between WT and HD neurons.

### Enzyme-linked immunosorbent assay (ELISA)

Released BDNF was measured using the mouse BDNF sandwich ELISA kit (Biomatik, Wilmington, DE). Neurons were incubated in 80 mM K^+^ for 10 min, and BDNF release was measured in accordance with the manufacturer’s instructions. Absorbance at 450 nm was read using a FlexStation 3 Multi-Mode Microplate Reader (Molecular Devices). Standard curves were prepared with 7.8–500 pg/ml BDNF. The amount of BDNF released was divided by the total amount of protein in each sample as described previously^37–39^.

### Soma size of striatal neurons

We treated both WT and HD primary striatal neurons with 50 ng/ml BDNF at DIV 5^9^. Two days later, we performed bright-field imaging of both treated and untreated neurons using a 100x objective. Somas were identified and were measured using ImageJ software as described previously^9^. Data were analyzed in a double-blind fashion.

### Quantitative RT-PCR

Neurons were harvested at DIV 14–17. Total RNA was isolated using TRIzol reagent (Ambion), and the integrity of the RNA was estimated using gel electrophoresis. cDNA was synthesized from 1 µl of DNase-treated RNA using the QuantiNova Reverse Transcription kit (Qiagen) and was used as a template for quantitative reverse transcription polymerase chain reaction (qRT-PCR). qRT-PCR was performed in triplicate using the LightCycler 480 SYBR Green I Master Mix system (Roche). The relative level of *BDNF* mRNA was normalized to *α-tubulin* mRNA, which served as an endogenous internal control. The following primers were used for RT-PCR: murine *BDNF* sense: CCGGTATCCAAAGGCCAACT and antisense: CTGCAGCCTTCCTTGGTGTA; mouse *α-tubulin* sense: TGGCTGCCCTAGAGAAGGAT and antisense: GGAAGCAGCACCTTGTGACAT.

### Antibodies

Immunoblotting was performed using the following antibodies: anti-phospho-TrkB (Tyr816; ABN1381; EMD Millipore Corporation, 1:500), anti-BDNF (N-20, sc546; Santa Cruz Biotechnology, Inc., 1:200), anti-alpha Tubulin (ab52866; Abcam; 1:1000), and anti-rabbit IgG HRP-conjugated (A0545; Sigma-Aldrich; 1:100,000). Protein band intensities were quantified using ImageJ.

### Immunoblotting

Neurons were lysed using N-PER Neuronal Protein Extraction Reagent (Thermo Scientific). Lysates were cleared by centrifugation for 21 min at 4 °C, boiled in sample buffer (120 mM Tris-HCl, 4% SDS, 20% glycerol, 5% beta-mercaptoethanol, and 0.1 mg bromophenol blue), resolved using SDS-PAGE, and transferred to a PVDF membrane. Membranes were blocked in 5% (w/v) dry milk or BSA in TBS-T (0.1% Tween-20 in TBS), incubated with primary antibodies overnight at 4 °C in TBS-T containing 3% BSA, washed with TBS-T, incubated with HRP-conjugated secondary antibodies at room temperature for 1 h, washed in TBS-T, and visualized using Clarity Western ECL Substrate (Bio-Rad Laboratories) with ChemiDoc (Bio-Rad Laboratories).

### Analysis

MetaMorph (Molecular Devices) was used to measure the fluorescence intensity within ROIs. The relative intensity of each ROI was calculated using custom-made MATLAB programs (MathWorks). All summary data are presented as the mean ± SEM (standard error of the mean). Where appropriate, differences between WT and HD neurons were analyzed using the non-parametric Mann-Whitney *U* test^40^. The proportion of event types between WT and HD neurons was compared using the Chi-square test^35^. Fluorescence intensities were fitted with the single exponential *∆f/f*_0_ = a*(1 − *e*^−*t*/*τ*^) + *c* using a custom-made MATLAB program to calculate the rise and decay time constants. All statistical analyses were conducted using IBM SPSS Statistics 19 software. Differences were considered significant at *p* < 0.05.

## Results

### Release of BDNF-pHluorin in cortical neurons

First, we tested whether BDNF secretion is altered in primary cortical neurons obtained from our zQ175 knock-in mouse model of HD, which closely mimics HD in humans in terms of genetic context and the late-onset, slow progression, and neuropathology^41,42^. Neurons were stimulated with 80 mM K^+^, and secreted BDNF was measured in the culture medium using ELISA. We found a significant decrease in secreted BDNF levels in HD cortical neurons compared to WT neurons (*p* = *0*.*0065*, Mann-Whitney *U* test, Supplementary Fig. S1).

Next, to investigate the mechanisms that underlie this decrease in BDNF secretion, we examined the exocytosis of BDNF in cortical neurons. We transfected cortical neurons with BDNF-pHluorin, a reporter in which BDNF is fused to pH-sensitive GFP (pHluorin)^27^. In transfected neurons, exocytosis is observed as a rapid increase in BDNF-pHluorin fluorescence. This construct was previously shown to function similar to endogenous BDNF, including TrkB receptor phosphorylation and activation of downstream signaling pathways^27^. BDNF-pHluorin fluorescence was initially quenched by the acidic environment in the lumen of BDNF-containing vesicles; however, fluorescence increased when the reporter is exposed to the external solution of pH ~7.3 upon opening of the fusion pore (Fig. 1A). BDNF release in cortical neurons was measured by performing 1-Hz time-lapse imaging at DIV 14–21. Neurons expressing BDNF-pHluorin were first identified by the application of NH_4_Cl, which caused a massive increase in fluorescence due to de-acidification (Supplementary Fig. S2A,B). In agreement with a previous report^27^, BDNF-containing vesicles exhibited distinct release patterns when various stimulation protocols were used (Supplementary Fig. S3). For our experiments, we used 300 field stimuli (10 Hz for 30 s), which does not induce plasticity and is close to physiological conditions, as *in vivo* neurons experience tonic activity ranging from 1 to 20 Hz^27,43,44^. Before stimulation, 20 frames were recorded as the baseline. Neurons were then stimulated using custom-made parallel electrodes, and after a brief resting period following the stimulation, neurons were perfused with NH_4_Cl (Fig. 1A). NH_4_Cl permeated into the lumen of BDNF-containing vesicles and de-quenched all BDNF-pHluorin in the presynaptic terminals (Fig. 1A). Thus, local expression of BDNF-pHluorin was estimated based on the sum of the peak intensity during stimulation and during NH_4_Cl perfusion. Relative intensities of BDNF-pHluorin determined during the stimulation and the NH_4_Cl perfusion were monitored independently in WT and HD neurons (Fig. 1B,C). Peak intensities obtained during electrical stimulation and during NH_4_Cl perfusion are represented as *I*_*1*_ and *I*_2_, respectively, and the peak intensity during stimulation (*I*_1_) was significantly lower in HD neurons than WT neurons (*p* = *0*.*00004427*, Mann-Whitney *U* test) (Fig. 1D,inset). The release ratio of BDNF-pHluorin during stimulation was calculated as the intensity ratio [*I*_1_*/(I*_1_ + *I*_2_)] (Fig. 1E) in order to reflect the variance in fluorescent puncta; the results show that the release ratio during stimulation was significantly lower in HD neurons than WT neurons (*p* = *0*.*0000003892*, Mann-Whitney *U* test, Fig. 1E). This indicates that BDNF release is markedly reduced in HD cortical neurons.

### Release of BDNF-pHluorin from cortical neurons projecting to striatal neurons

**Figure 2:**
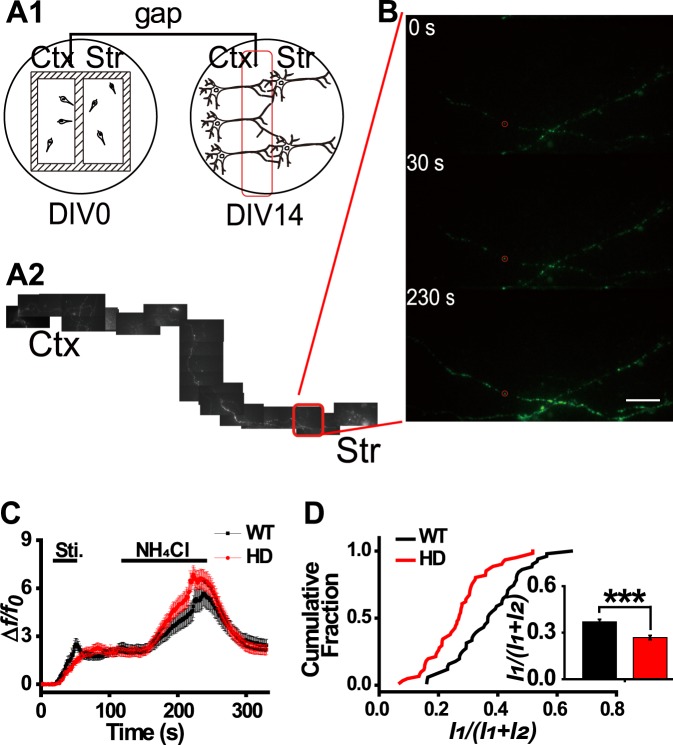
Release of BDNF-pHluorin from cortical projection neurons close to striatal neurons. (**A**) Schematic of corticostriatal co-cultures prepared using ibidi 2-well culture inserts and a representative image. (A1) Cortical and striatal neurons were plated in separate compartments and they formed connections after the inserts were removed. A gap area close to the striatal compartment is marked in red. (A2) Representative image showing that a cortical neuron projected to the striatal compartment, in which the imaging area is highlighted by the red box. (**B**) Response at different times of the cortical projection neuron shown in (A2). (**C**) Relative fluorescence intensity traces of BDNF-pHluorin at cortical presynaptic terminals close to striatal neurons. (**D**) Cumulative plot of peak intensity ratio in WT and HD neurons. Inset, bar graph of intensity ratio, showing a significant decrease in HD neurons (n = 61) as compared with that in WT neurons (n = 51); ^***^p < 0.001, Mann-Whitney *U* test. Scale bar: 10 μm.

To test whether BDNF release is decreased in cortical neurons projecting to striatal neurons, we co-cultured cortical and striatal neurons using ibidi 2-well culture inserts (Fig. 2A); the inserts were removed at DIV 1, after which the cortical neurons could project freely into the striatal compartment (Fig. 2A). To verify the formation of corticostriatal connections, striatal neurons were electroporated with a construct expressing the glutamate sensor iGluSnFR; the fluorescence intensity of this sensor increases upon binding glutamate^28^. Because the striatum primarily contains inhibitory neurons, the glutamate captured in the striatal neurons in our co-culture is likely derived from cortical projection neurons. Cortical presynaptic terminals were identified based on the spontaneous loading of FM4–64. After washing to eliminate nonspecific binding, field stimulation was applied (Supplementary Fig. S4A). The iGluSnFR signal increased rapidly after stimulation in regions that were near FM4-64 puncta (e.g., the red ROI) but increased slowly in regions far from the FM4-64 puncta (e.g., the blue ROI) (Supplementary Fig. S4A,B). These results indicate that active connections were formed between the co-cultured cortical and striatal neurons.

Neurons isolated from the cortex were electroporated with the BDNF-pHluorin construct before being plated into dishes with the 2-well inserts; the axons from these cortical neurons that projected into the compartments containing striatal neurons were then imaged (Fig. 2A). The same stimulation protocol was applied to WT and HD neurons, and total fluorescence was measured during stimulation and during NH_4_Cl perfusion (Fig. 2B,C). As in the case of pure cortical HD neurons, BDNF release from the co-cultured HD cortical neurons was decreased relative to WT neurons. The intensity ratio determined for the HD cortical axons that projected into striatal compartments was significantly lower than WT axons (*p* = *0*.*00000638*1, Mann-Whitney *U* test) (Fig. 2D, inset). Thus, we conclude that BDNF release is decreased in co-cultured cortical neurons from zQ175 mice.

### Exocytosis of single BDNF-pHluorin-containing vesicles

To investigate whether the decreased BDNF release was caused by differences in the prevalence of the specific release patterns of BDNF-containing vesicles, we measured BDNF release from single BDNF-containing vesicles using total internal reflection fluorescence microscopy (TIRFM) during 300 electrical stimuli (50 Hz for 6 s). For these experiments, we used a higher stimulation frequency, as this frequency is more effective at triggering the release of BDNF-containing vesicles compared to 10 Hz stimulation^35,45,46^. Because TIRFM excites fluorescent molecules selectively near the cell surface, this technique can be used to observe the release of single BDNF containing vesicles^47–49^. We observed the exocytosis of single BDNF-containing vesicles similar to the previously measured exocytosis from single synaptic vesicles^47^. Under TIRFM, the increase in fluorescence intensity is caused by the de-acidification that occurs after fusion-pore opening near the surface of the coverslip. To confirm that the fluorescence increase in BDNF-pHluorin observed under TIRFM was caused by opening of the fusion pore and de-acidification of vesicles, we applied the membrane-impermeable MES buffer after electrical stimulation^35^. All events were quenched by MES (Supplementary Fig. S5A), which indicates that the fluorescence changes represented opening of the fusion pore of BDNF-containing vesicles during stimulation. Because the decay in the fluorescence intensity of BDNF-pHluorin could have been caused by vesicle re-acidification, we measured the effect of re-acidification on fluorescence changes in BDNF-pHluorin^27,31^ using the vesicular H^+^-ATPase inhibitor bafilomycin^35^. Application of bafilomycin did not affect the time course of BDNF-pHluorin release, as the decay time constants measured in the presence and absence of bafilomycin did not differ significantly (*p* = 0.5667, Mann-Whitney *U* test) (Supplementary Fig. S5C). In agreement with previous reports^35,45^, our results indicate that the release of single BDNF-containing vesicles is adequately detected as a change in BDNF-pHluorin fluorescence under TIRFM.

**Figure 3:**
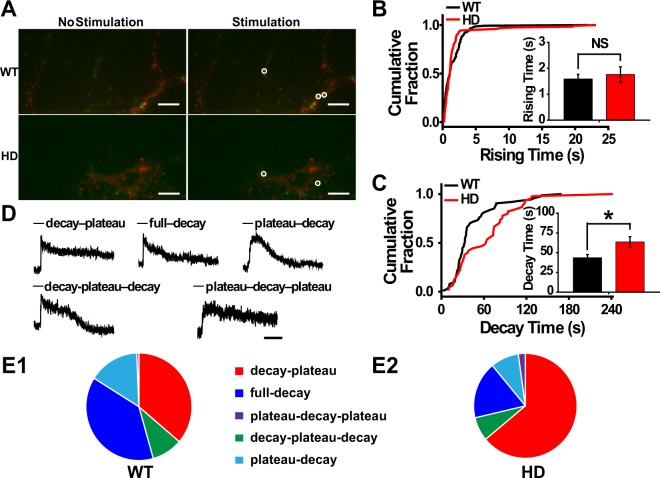
Release patterns of single BDNF-pHluorin-containing vesicles in cortical projection neurons close to striatal neurons. (**A**) Representative images of single BDNF-containing vesicles (green) before (left) and after (right) stimulation. Presynaptic terminals are identified by FM4-64 puncta (red). Arrows indicate the release of single BDNF-containing vesicles. (**B**) Cumulative plot of the rising time constant of fluorescence intensity, which did not differ significantly between WT and HD neurons (Inset, *p* = 0.8075, Mann-Whitney *U* test). (**C**) Cumulative plot of decay time constant, which differed significantly between WT and HD neurons (inset, *p* = *0*.*01474*, Mann-Whitney *U* test). (**D**) Exemplar traces of 5 release patterns. (**E**) Proportions of distinct release patterns. As compared to WT (n = 149 events, **E1**), HD neurons (n = 136 events, **E2**) showed a reduction in the fraction of “full-decay” events but an increase in that of “decay-plateau” events (*p* < 0.0001, paired χ^2^ test). NS, not statistically significant, and ^*^p < 0.05, Mann-Whitney *U* test. Scale bar: 10 μm.

Next, we measured the exocytosis of single BDNF-pHluorin-containing vesicles in WT and HD cortical neurons co-cultured with striatal neurons. Cortical neurons were electroporated with the BDNF-pHluorin construct, and presynaptic terminals were identified based on FM4-64-labeled synaptic vesicles (Fig. 3A). We found that the rise time constant was similar between WT and HD neurons (*p* = 0.8075, Mann-Whitney *U* test) (Fig. 3B); in contrast, the decay time constant was significantly increased in HD neurons (*p* = *0*.*01474*, Mann-Whitney *U* test) (Fig. 3C). Consistent with a previous report^35^, we observed diverse release patterns for single BDNF-pHluorin-containing vesicles, even within the same neuron (Fig. 3D); we classified each of these release events into 5 groups based on their kinetics^35^. Most of the release events were “fast-decay” events that occurred immediately after the fluorescence reached peak intensity, and these events were further divided into the following 3 groups: “full-decay,” “decay-plateau,” and “decay-plateau-decay.” The “full-decay” group included events in which the fluorescence intensity decreased to baseline levels, suggesting full release or the persistence of a dilated fusion pore for single BDNF-pHluorin-containing vesicles^35^. In contrast, the “decay-plateau” group included events in which the intensity remained above baseline until the end of the imaging experiment, suggesting transient opening of the fusion pore for single BDNF-pHluorin-containing vesicles, similar to the release of synaptic vesicles^35,50^. In certain cases of full decay, the decay was interrupted by a brief plateau, and these events were categorized as “decay-plateau-decay” release events. In the remainder of the events, fluorescence intensity was maintained at the peak level briefly before decay, and these were named “plateau-decay” or “plateau-decay-plateau” events depending on whether the intensity reached baseline by the final imaging frame (Fig. 3D). We found that the proportions of these release patterns differed significantly between WT and HD neurons (*p* = 0.000036, χ^2^ test) (Fig. 3E). Specifically, more “decay-plateau” events were observed in HD neurons compared to WT neurons (63.97% vs. 36.24%, respectively; *p* = 0.0000029, paired χ^2^ test), whereas “full-decay” events were significantly less prevalent in HD neurons compared to WT neurons (17.65% vs. 38.26%, respectively; *p* = 0.00012, paired χ^2^ test); however, the prevalence of both “plateau-decay” events (*p* = 0.089, paired χ^2^ test) and “decay-plateau-decay” (*p* = 0.54, paired χ^2^ test) events were similar between HD and WT neurons. Our results reveal a significant change in the release pattern of BDNF-containing vesicles in HD neurons, leading to a decrease in the amount of BDNF released in HD neurons.

### Transport of BDNF in cortical neurons

**Figure 4:**
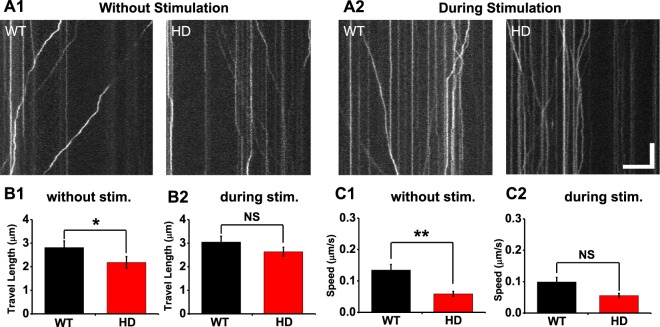
Transport of BDNF-EGFP-containing vesicles in cultured cortical neurons. (**A**) Representative kymographs of BDNF-EGFP-containing vesicles without (A1) and during (A2) stimulation of WT and HD neurons. (**B**) Total travel length for BDNF-GFP-containing vesicles in without (B_1_, n = 145 for WT; n = 149 for HD) and during (B_2_, n = 114 for WT; n = 148 for HD) field stimulation. (**C**) Speed of BDNF-EGFP-containing vesicles in WT and HD neurons without (C_1_) and during (C_2_) stimulation. ^*^p < 0.05, Mann-Whitney *U* test; NS, not statistically significant. Scale bar: 10 µm, 5 s.

Because the axonal transport of BDNF from the soma to presynaptic terminals in cortical neurons can also affect the delivery of BDNF to neurons in the striatum, we examined whether the axonal transport of BDNF-containing vesicles is altered in primary cortical neurons cultured from zQ175 mice. To measure the transport of BDNF-containing vesicles, we transfected cortical neurons with BDNF-EGFP and performed real-time imaging at 10 Hz; these images were then used to measure the total travel length and speed of BDNF-containing vesicles moving along the axons. Figure 4A shows the kymographs of BDNF-EGFP-containing vesicles moving along the axons of HD and WT cortical neurons, showing both anterograde and retrograde movement of BDNF-containing vesicles. The total travel length and speed of BDNF-EGFP-containing vesicles moving along axons judged by the morphology were calculated with the ImageJ macro Kymolyzer^36^. Under basal conditions, both the total travel length and vesicle speed were significantly lower in HD neurons compared to WT neurons (*p* = *0*.*03999* and *p* = *0*.*003691* respectively, Mann-Whitney *U* test) (Fig. 4B1,C1). This impaired movement of vesicles in HD neurons suggests a deficit in the transport of BDNF-containing vesicles, which may reduce the delivery of BDNF from cortical neurons to neurons in the striatum.

We also examined whether the transport of BDNF-containing vesicles in HD cortical neurons during activity induced by 300 electrical stimuli is altered in comparison with WT neurons. Figure 4A2 shows the kymographs of BDNF-EGFP-containing vesicles during stimulation, showing both anterograde and retrograde movement. In contrast to our results obtained under basal conditions, we found that neither the total travel length nor the speed of movement differed significantly between HD and WT cortical neurons during field stimulation (*p* = 0.3645 and *p* = 0.1053, respectively; Mann-Whitney *U* test) (Fig. 4B2,C2). These results indicate that the transport of BDNF-containing vesicles during the external stimulation is not altered in HD cortical neurons compared with that of WT neurons.

### Treating striatal neurons with BDNF

**Figure 5:**
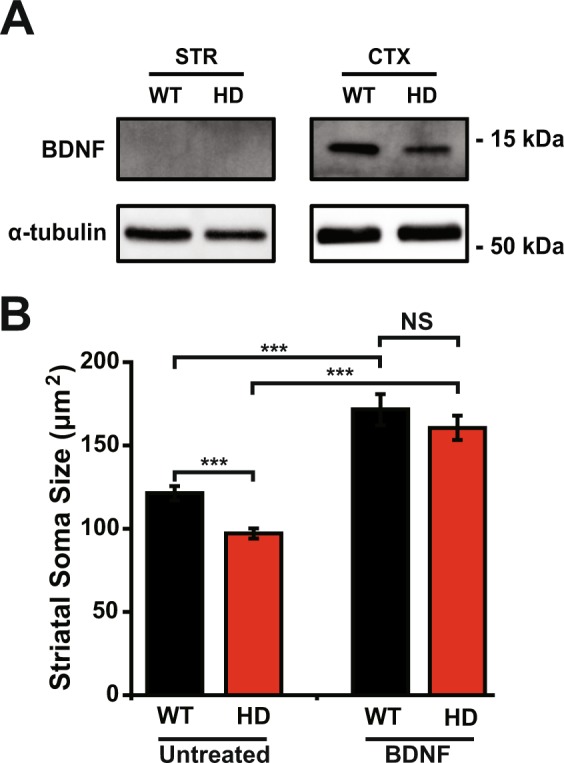
BDNF treatment increases the soma size of striatal neurons. (**A**) Immunoblot analysis of lysates from pure primary striatal (STR) and cortical (CTX) neurons at DIV 14 using an anti-BDNF antibody. (**B**) Soma size of untreated and BDNF-treated primary striatal neurons. NS, not statistically significant; ^***^p < 0.001, Mann-Whitney *U* test.

Next, we used western blot analysis to measure BDNF levels in both striatal and cortical neurons. Our analysis revealed that cortical neurons produce robust levels of BDNF, whereas striatal neurons do not express measurable levels of BDNF (Fig. 5A). Thus, striatal neurons do not express relevant levels of BDNF and likely receive BDNF from the cortex^19,22^.

Given this key finding, we then examined whether the changes in striatal neurons in zQ175 mice due to decreased delivery of BDNF from cortical neurons can be prevented by applying BDNF to striatal neurons. We treated striatal neurons at DIV 5 with 50 ng/ml BDNF for two days and then measured soma size using ImageJ analysis of bright-field images. We found that the soma size of untreated HD striatal neurons was significantly smaller than untreated WT striatal neurons (*p* = *0*.*000023*, Mann-Whitney *U* test) (Fig. 5B). Treating both HD and WT neurons with BDNF significantly increased their soma size (*p* = 0.00000015 for WT striatal neurons and *p* = *2*.8*10^*−10*^ for HD striatal neurons, Mann-Whitney *U* test). Interestingly, after BDNF treatment, soma size was no longer significantly different between HD and WT neurons (*p* = 0.35, Mann-Whitney *U* test). These results indicate that treating striatal HD neurons with BDNF can prevents changes in cellular morphology, suggesting a possible new therapeutic approach to treating HD patients.

## Discussion

In this study, we report the real-time measurement of release and transport of BDNF in cultured cortical neurons using a zQ175 knock-in mice, as model for HD. We found that BDNF release is markedly decreased in both the cortical neurons and their projections to co-cultured striatal neurons in zQ175 mice. We also observed distinct release patterns of single BDNF-containing vesicles; specifically, the proportion of “full-decay” events was considerably lower in HD neurons compared to WT neurons. In addition, we found that the axonal transport of BDNF is significantly impaired in zQ175 cortical neurons.

Our findings suggest that the delivery of BDNF from the cortex to the striatum is impaired in HD. This impaired delivery of BDNF may underlie the high vulnerability of striatal neurons in HD, as BDNF plays an essential role in promoting neuronal survival, maturation, and synaptic activity^15–18^. Striatal neurons produce little BDNF^19–21^ and receive BDNF largely from the cortex though they were reported to receive BDNF from other brain regions including the thalamus and mesencephalon^19,22,51^. Our findings therefore provide a plausible explanation for the recent report that presynaptic dysfunction causes pathological features in the striatal compartment in an *in vitro* corticostriatal network in HD^52^. Although our findings are based on cultured neurons from newborn mice, which may not necessarily reflect the *in vivo* situation in patients, our results are consistent with a recent study using acute striatal slices from HD mice, which found altered cortical secretion of BDNF^53^.

We observed the significant reduction of the soma size in untreated HD striatal neurons compared with untreated WT striatal neurons. The similar results were reported in a previous study using striatal neurons cultured from BACHD mice (another mouse model of HD)^9^. However, no significant reduction of the soma size was observed in the brain slice from 2-month old HD mice^54^. Future work is needed to address this difference between cultured striatal neurons and the brain slices from young HD mice.

Our finding that the prevalence of “full-decay” and “decay-plateau” release patterns are decreased and increased, respectively, in zQ175 cortical neurons suggests a possible mechanism underlying the reduced release of BDNF in HD cortical neurons. Thus, a decrease in cortical BDNF release would result in reduced corticostriatal synaptic activity, which would further decrease BDNF release due to weakened synaptic strength^55–57^, ultimately leading to the degeneration of striatal neurons as the disease progresses. Nevertheless, other mechanisms may contribute to the degeneration of striatal neurons in HD. For example, several groups reported altered TrkB receptor signaling in response to BDNF in HD^40,51,58^. In addition, the p75 neurotrophin receptor (p75NTR), which binds BDNF with low affinity, was suggested to play a role in the pathophysiology of HD^40,59^; moreover, synaptic plasticity was rescued by inhibiting either p75NTR signaling or its downstream pathway^40^. Thus, p75NTR signaling may play a role in the degeneration of striatal neurons in HD, although the role that p75NTR signaling plays in striatal neurons may change during development. Further studies are clearly warranted in order to identify the detailed mechanisms underlying the degeneration of striatal neurons in HD.

The molecular mechanisms that underlie the reduction in BDNF release in HD are poorly understood. However, our findings suggest a possible interaction between the mutant huntingtin protein and the release machinery in dense-core vesicles. For example, the increased prevalence of transient fusion pore openings suggests a possible interaction between the mutant huntingtin protein and Huntingtin-associated protein 1 (HAP1) during exocytosis, as ΗΑP1 is localized to synaptic vesicles^60^ and dense-core vesicles in neurons^61^, and loss of HAP1 was reported to decrease the number of full-fusion event by regulating the stability of fusion pore opening in dense-core vesicles^62^. The mutant huntingtin protein may therefore alter fusion pore opening by altering the normal function of HAP1; this notion is supported by the finding that the mutant huntingtin protein binds to HAP-1 more strongly than the wild-type huntingtin protein^63^. In addition, α-synuclein may play a role in the increased prevalence of transient fusion pore opening in HD, given that α-synuclein promotes fusion pore dilation in dense-core vesicles during exocytosis^35^. Because the release of BDNF is believed to be modulated by a combination of distinct release mechanisms^10,35^, the putative interactions between the mutant huntingtin protein and other proteins such as SNAP25, SNAP47, and synaptotagmin-4 warrant investigation. In addition, further study is needed in order to identify the molecular mechanisms that underlie the decreased release of BDNF in HD; such studies will also help elucidate the mechanisms that underlie striatal neurodegeneration as the disease progresses.

In conclusion, we report decreased release and transport of BDNF in cortical neurons in zQ175 mice. In addition, we show that the prevalence of “full-decay” release events of single BDNF-containing vesicles is reduced in HD neurons, which may explain the decreased BDNF release observed at single synaptic terminals. Thus, our findings provide insight into the impaired delivery of BDNF in HD and suggest a possible pathogenic mechanism underlying the degeneration of striatal neurons in HD. Finally, our measurements of the exocytosis and transport of BDNF-containing vesicles may help facilitate the development of new therapeutic approaches to HD.

## Electronic supplementary material

Supplementary Dataset 1

## Data Availability

The datasets generated during and/or analyzed during the current study are available from the corresponding author on reasonable request.

## References

[1] MacDonald ME (1993). A novel gene containing a trinucleotide repeat that is expanded and unstable on Huntington’s disease chromosomes. Cell.

[2] Romero E (2008). Suppression of neurodegeneration and increased neurotransmission caused by expanded full-length huntingtin accumulating in the cytoplasm. Neuron.

[3] Zuccato C, Valenza M, Cattaneo E (2010). Molecular mechanisms and potential therapeutical targets in Huntington’s disease. Physiol. Rev..

[4] Zuccato C, Cattaneo E (2014). Huntington’s disease. Handb. Exp. Pharmacol..

[5] Tsoi H, Chan HY (2013). Expression of expanded CAG transcripts triggers nucleolar stress in Huntington’s disease. Cerebellum.

[6] Walker FO (2007). Huntington’s disease. Lancet.

[7] Yu S, Liang Y, Palacino J, Difiglia M, Lu B (2014). Drugging unconventional targets: insights from Huntington’s disease. Trends Pharmacol. Sci..

[8] Garcia-Miralles M (2016). Laquinimod rescues striatal, cortical and white matter pathology and results in modest behavioural improvements in the YAC128 model of Huntington disease. Sci. Rep..

[9] Zhao X (2016). TRiC subunits enhance BDNF axonal transport and rescue striatal atrophy in Huntington’s disease. Proc. Natl. Acad. Sci. USA.

[10] Lewin GR, Carter BD (2014). Neurotrophic factors. Preface. Handb. Exp. Pharmacol..

[11] Zuccato C, Cattaneo E (2009). Brain-derived neurotrophic factor in neurodegenerative diseases. Nat. Rev. Neurol..

[12] Won H, Mah W, Kim E (2013). Autism spectrum disorder causes, mechanisms, and treatments: focus on neuronal synapses. Front. Mol. Neurosci..

[13] Lee R, Kermani P, Teng KK, Hempstead BL (2001). Regulation of cell survival by secreted proneurotrophins. Science.

[14] Woo NH (2005). Activation of p75NTR by proBDNF facilitates hippocampal long-term depression. Nat. Neurosci..

[15] Zuccato C, Cattaneo E (2007). Role of brain-derived neurotrophic factor in Huntington’s disease. Prog. Neurobiol..

[16] Lu B (2003). BDNF and activity-dependent synaptic modulation. Learn. Mem..

[17] Acheson A (1995). A BDNF autocrine loop in adult sensory neurons prevents cell death. Nature.

[18] Huang EJ, Reichardt LF (2001). Neurotrophins: roles in neuronal development and function. Annu. Rev. Neurosci..

[19] Altar CA (1997). Anterograde transport of brain-derived neurotrophic factor and its role in the brain. Nature.

[20] Hofer M, Pagliusi SR, Hohn A, Leibrock J, Barde YA (1990). Regional distribution of brain-derived neurotrophic factor mRNA in the adult mouse brain. EMBO J..

[21] Schmidt-Kastner R, Wetmore C, Olson L (1996). Comparative study of brain-derived neurotrophic factor messenger RNA and protein at the cellular level suggests multiple roles in hippocampus, striatum and cortex. Neuroscience.

[22] Baquet ZC, Gorski JA, Jones KR (2004). Early striatal dendrite deficits followed by neuron loss with advanced age in the absence of anterograde cortical brain-derived neurotrophic factor. J. Neurosci..

[23] Zuccato C (2001). Loss of huntingtin-mediated BDNF gene transcription in Huntington’s disease. Science.

[24] Cattaneo E, Zuccato C, Tartari M (2005). Normal huntingtin function: an alternative approach to Huntington’s disease. Nat. Rev. Neurosci..

[25] Ferrer I, Goutan E, Marín C, Rey MJ, Ribalta T (2000). Brain-derived neurotrophic factor in Huntington disease. Brain Res..

[26] Randall FE (2011). The corticostriatal system in dissociated cell culture. Front. Syst. Neurosci..

[27] Matsuda N (2009). Differential activity-dependent secretion of brain-derived neurotrophic factor from axon and dendrite. J. Neurosci..

[28] Marvin JS (2013). An optimized fluorescent probe for visualizing glutamate neurotransmission. Nat. Methods.

[29] Park H, Li Y, Tsien RW (2012). Influence of synaptic vesicle position on release probability and exocytotic fusion mode. Science.

[30] Alsina A (2017). Real-time subpixel-accuracy tracking of single mitochondria in neurons reveals heterogeneous mitochondrial motion. Biochem. Biophys. Res. Commun..

[31] Hartmann D, Drummond J, Handberg E, Ewell S, Pozzo-Miller L (2012). Multiple approaches to investigate the transport and activity-dependent release of BDNF and their application in neurogenetic disorders. Neural Plast..

[32] Chung C, Barylko B, Leitz J, Liu X, Kavalali ET (2010). Acute dynamin inhibition dissects synaptic vesicle recycling pathways that drive spontaneous and evoked neurotransmission. J. Neurosci..

[33] Wilhelm BG, Groemer TW, Rizzoli SO (2010). The same synaptic vesicles drive active and spontaneous release. Nat. Neurosci..

[34] Oh WC, Lutzu S, Castillo PE, Kwon HB (2016). De novo synaptogenesis induced by GABA in the developing mouse cortex. Science.

[35] Logan T, Bendor J, Toupin C, Thorn K, Edwards RH (2017). α-Synuclein promotes dilation of the exocytotic fusion pore. Nat. Neurosci..

[36] Pekkurnaz G, Trinidad JC, Wang X, Kong D, Schwarz TL (2014). Glucose regulates mitochondrial motility via Milton modification by O-GlcNAc transferase. Cell.

[37] Canals JM (2004). Brain-derived neurotrophic factor regulates the onset and severity of motor dysfunction associated with enkephalinergic neuronal degeneration in Huntington’s disease. The Journal of neuroscience.

[38] Stansfield KH, Bichell TJ, Bowman AB, Guilarte TR (2014). BDNF and Huntingtin protein modifications by manganese: implications for striatal medium spiny neuron pathology in manganese neurotoxicity. J Neurochem.

[39] Hong Y, Zhao T, Li X-J, Li S (2016). Mutant Huntingtin Impairs BDNF Release from Astrocytes by Disrupting Conversion of Rab3a-GTP into Rab3a-GDP. The Journal of Neuroscience.

[40] Plotkin JL (2014). Impaired TrkB receptor signaling underlies corticostriatal dysfunction in Huntington’s disease. Neuron.

[41] Heikkinen T, *et al*, Characterization of neurophysiological and behavioral changes, MRI brain volumetry and 1H MRS in zQ175 knock-in mouse model of Huntington’s disease. Plos One (2012). 10.1371/journal.pone.0050717 PMC3527436 23284644

[42] Menalled Liliana B., Kudwa Andrea E., Miller Sam, Fitzpatrick Jon, Watson-Johnson Judy, Keating Nicole, Ruiz Melinda, Mushlin Richard, Alosio William, McConnell Kristi, Connor David, Murphy Carol, Oakeshott Steve, Kwan Mei, Beltran Jose, Ghavami Afshin, Brunner Dani, Park Larry C., Ramboz Sylvie, Howland David (2012). Comprehensive Behavioral and Molecular Characterization of a New Knock-In Mouse Model of Huntington’s Disease: zQ175. PLoS ONE.

[43] Bland BH (1986). The physiology and pharmacology of hippocampal formation theta rhythms. Progress in neurobiology.

[44] Larson J, Munkacsy E, Theta-burst LTP (1621). Brain research.

[45] Shimojo M (2015). SNAREs controlling vesicular release of BDNF and development of callosal axons. Cell Rep..

[46] van de Bospoort R (2012). Munc13 controls the location and efficiency of dense-core vesicle release in neurons. The Journal of cell biology.

[47] Yu C, Zhang M, Qin X, Yang X, Park H (2016). Real-time imaging of single synaptic vesicles in live neurons. Front. Biol..

[48] Axelrod D (2001). Selective imaging of surface fluorescence with very high aperture microscope objectives. Journal of biomedical optics.

[49] Park H, Toprak E, Selvin PR (2007). Single-molecule fluorescence to study molecular motors. Quarterly reviews of biophysics.

[50] Gandhi SP, Stevens CF (2003). Three modes of synaptic vesicular recycling revealed by single-vesicle imaging. Nature.

[51] Nguyen, K Q., Vladimir, V. R & Sadikot, A F. Impaired TrkB Signaling Underlies Reduced BDNF-Mediated Trophic Support of Striatal Neurons in the R6/2 Mouse Model of Huntington’s Disease. *Frontiers in Cellular Neuroscience* 10 (2016). 10.3389/fncel.2016.00037 PMC4783409 27013968

[52] Virlogeux A (2018). Reconstituting Corticostriatal Network on-a-Chip Reveals the Contribution of the Presynaptic Compartment to Huntington’s Disease. Cell reports.

[53] Park H (2018). Cortical Axonal Secretion of BDNF in the Striatum Is Disrupted in the Mutant-huntingtin Knock-in Mouse Model of Huntington’s Disease. Experimental neurobiology.

[54] Indersmitten T (2015). “Altered excitatory and inhibitory inputs to striatal medium-sized spiny neurons and cortical pyramidal neurons in the Q175 mouse model of Huntington’s disease.”. Journal of Neurophysiology.

[55] Park H, Popescu A, Poo MM (2014). Essential role of presynaptic NMDA receptors in activity-dependent BDNF secretion and corticostriatal LTP. Neuron.

[56] Lu H, Park H, Poo MM (2014). Spike-timing-dependent BDNF secretion and synaptic plasticity. Philos. Trans. R. Soc. Lond. B. Biol. Sci..

[57] Guo W, Ji Y, Wang S, Sun Y, Lu B (2014). Neuronal activity alters BDNF–TrkB signaling kinetics and downstream functions. J. Cell Sci..

[58] Ginés Silvia, Paoletti Paola, Alberch Jordi (2010). Impaired TrkB-mediated ERK1/2 Activation in Huntington Disease Knock-in Striatal Cells Involves Reduced p52/p46 Shc Expression. Journal of Biological Chemistry.

[59] Brito, V. *et al*. Imbalance of p75NTR/TrkB protein expression in Huntington’s disease: implication for neuroprotective therapies. Cell Death & Disease. 2013. 10.1038/cddis.2013.116 PMC3641339 23598407

[60] Li SH, Li H, Torre ER, Li XJ (2000). Expression of huntingtin-associated protein-1 in neuronal cells implicates a role in neuritic growth. Molecular and cellular neurosciences.

[61] Wu LL, Fan Y, Li S, Li XJ, Zhou XF (2010). Huntingtin-associated protein-1 interacts with pro-brain-derived neurotrophic factor and mediates its transport and release. The Journal of biological chemistry.

[62] Mackenzie KD (2014). Huntingtin-associated protein 1 regulates exocytosis, vesicle docking, readily releasable pool size and fusion pore stability in mouse chromaffin cells. The Journal of physiology.

[63] Li XJ (1995). A huntingtin-associated protein enriched in brain with implications for pathology. Nature.

