# Supplementary material for: Decreased BDNF Release in Cortical Neurons of a Knock-in Mouse Model of Huntington’s Disease
Source: Sci Rep. 2018 Nov 19;8:16976. doi: 10.1038/s41598-018-34883-w (PMC6242964; doi:10.1038/s41598-018-34883-w)
Supplement: Supplementary file 1 — Supplementary Dataset 1 [file 41598_2018_34883_MOESM1_ESM.pdf]

Supplementary Figures

Decreased BDNF Release in Cortical Neurons of a Knock-in Mouse Model of Huntington's Disease

Chenglong Yu[1], Chun Hei Li[1], Sidong Chen[1], Hanna Yoo[1], Xianan Qin[2], and Hyokeun Park[1,2,3,*]

[1]Division of Life Science, [2]Department of Physics, and [3]State Key Laboratory of Molecular Neuroscience

The Hong Kong University of Science and Technology, Clear Water Bay, Kowloon, Hong Kong

***Corresponding Author**

Hyokeun Park PhD

Division of Life Science and Department of Physics

Hong Kong University of Science and Technology

Clear Water Bay

Kowloon, Hong Kong

Telephone: (852) 2358-7322

Fax: (852) 2358-1552

E-mail: hkpark@ust.hk

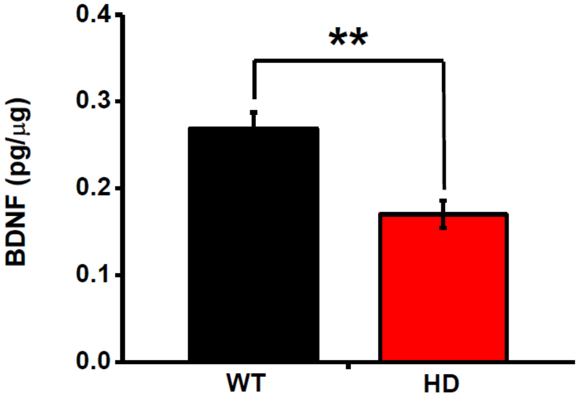

Supplementary Figure S1. ELISA was used to measure BDNF released following stimulation with 80 mM K^+. Cultured cortical neurons were stimulated with 80 mM K^+ for 10 min, and the supernatant was collected for ELISA (N=6 each). Total protein level was measured using the BCA method, and BDNF levels were normalized to total protein level. (**p<0.01, Mann-Whitney *U* test).

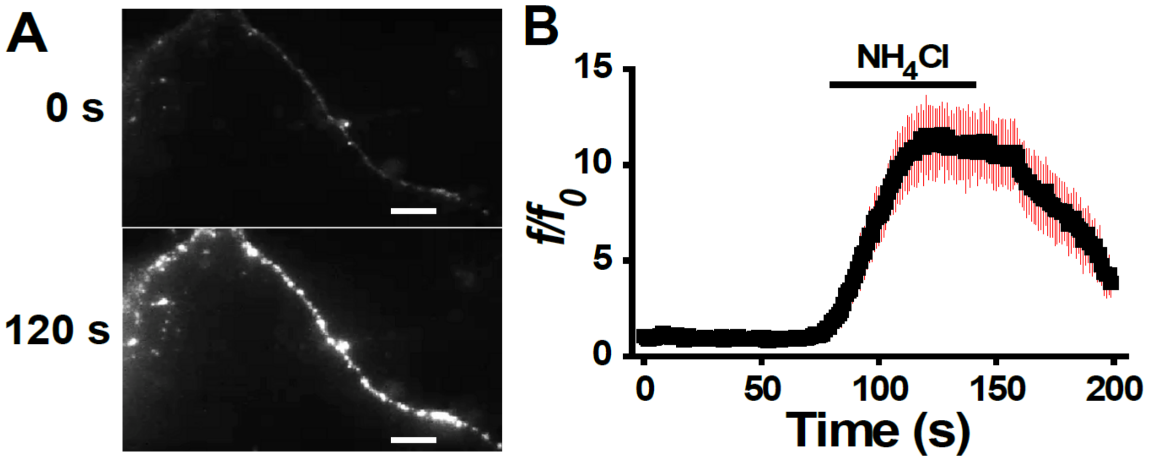

Supplementary Figure S2. Identification of BDNF-pHluorin transfected into cortical neurons. (**A**) Representative images of cortical axons transfected with BDNF-pHluorin and their response before (top, 0 s) and after (bottom, 120 s) NH_4Cl perfusion. (**B**) A relative fluorescence intensity trace of BDNF-pHluorin after NH_4Cl perfusion in puncta shown in (A). Scale bar: 10 µm.

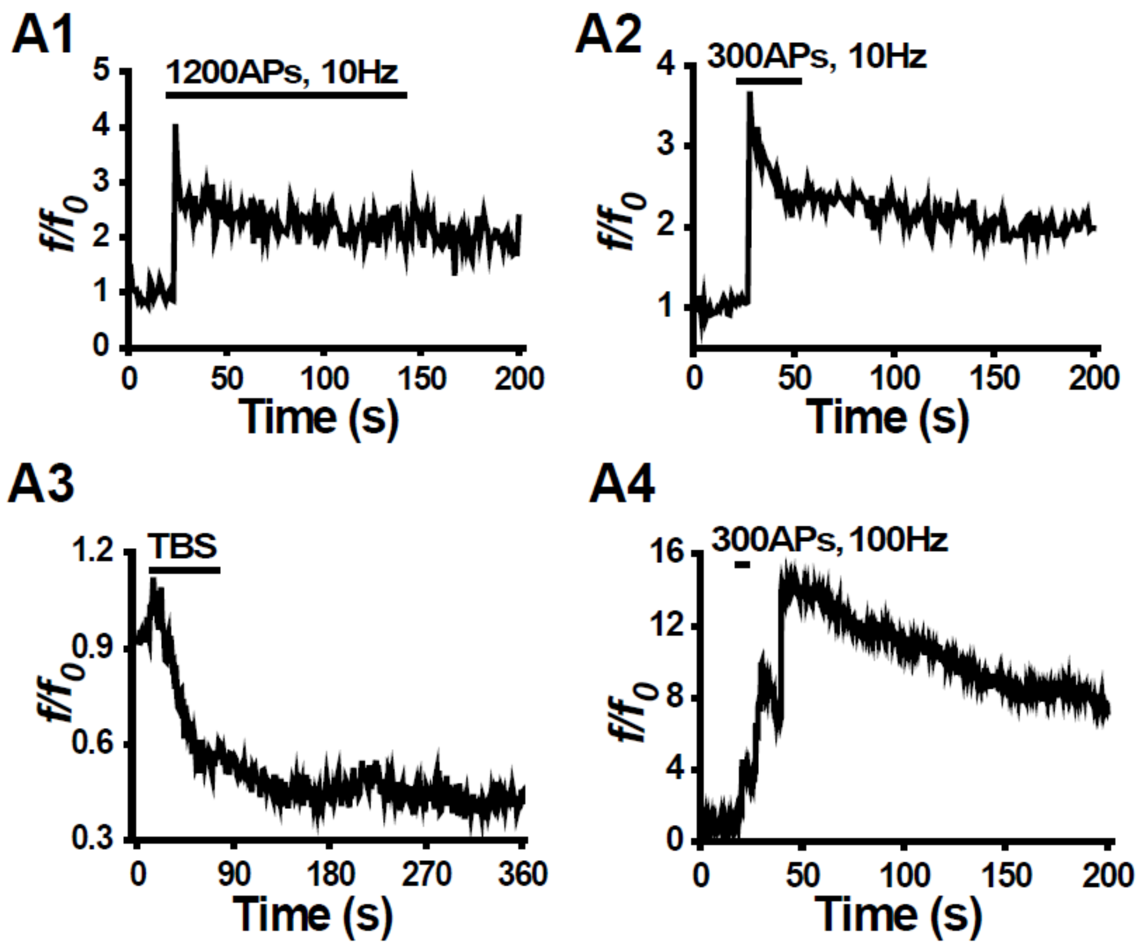

Supplementary Figure S3. Exemplar traces of BDNF-pHluorin release after different stimulation protocols. TBS, theta-burst stimulation, which contains 10 trains of stimuli with 5 s interval, and each train is composed by 10 pulses at 5 Hz with 4 spikes at 100 Hz.

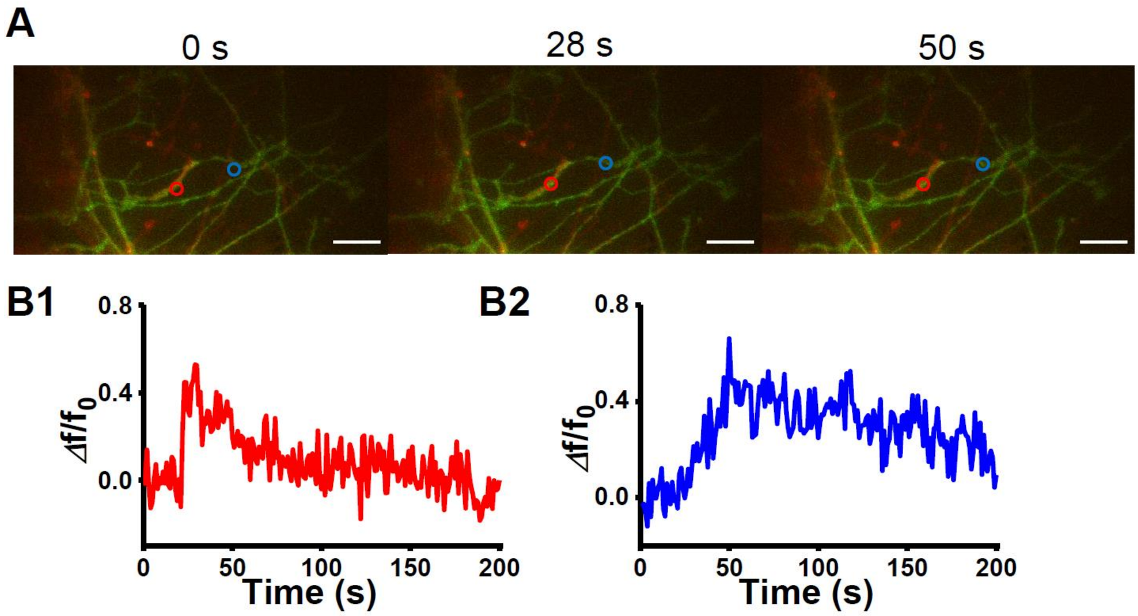

Supplementary Figure S4. Connection of cortical projection neurons to striatal neurons. (**A**) Representative images of iGluSnFR (green) response at different times during the electrical stimulation in co-cultured striatal neurons. Cortical presynaptic terminals were labeled by FM4-64 puncta (red). (**B**) Relative intensity traces of iGluSnFR at different ROIs marked red or blue shown in (A). Scale bar: 10 µm.

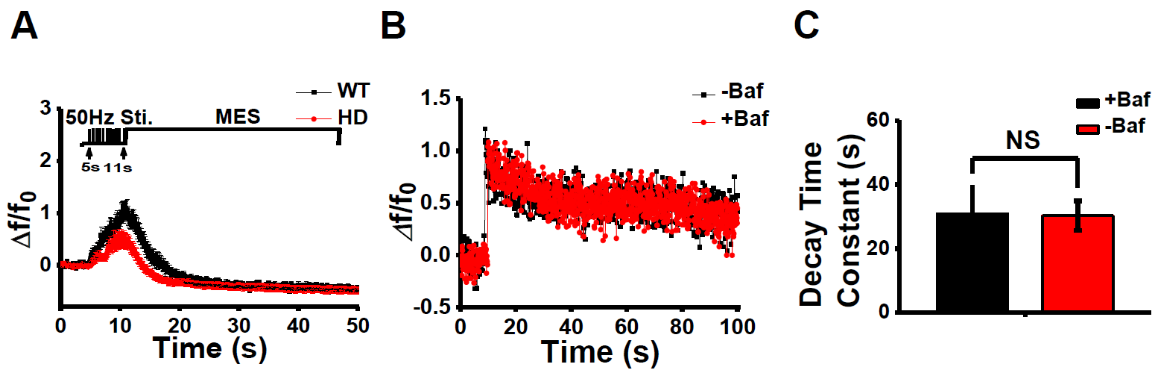

Supplementary Figure S5. Release of single BDNF-containing vesicles in cortical projections. (**A**) Average relative fluorescence intensity trace of BDNF-pHluorin, which were all quenched by MES perfusion. This quenching indicates that the increase of fluorescence is caused by the opening of fusion pore after stimulation. The average in WT traces contains 27 release events whereas the average in HD traces contains 23 release events. (**B**) Relative fluorescence traces of BDNF release with or without bafilomycin (a vesicular H^+-ATPase blocker). (**C**) A bar graph of decay time constant. Decay time constant of release events did not shows any significant difference with (n = 18) or without (n = 26) bafilomycin, indicating re-acidification do not affect the time course of BDNF-pHluorin release. (NS, not statistically significant, Mann-Whitney *U* test).

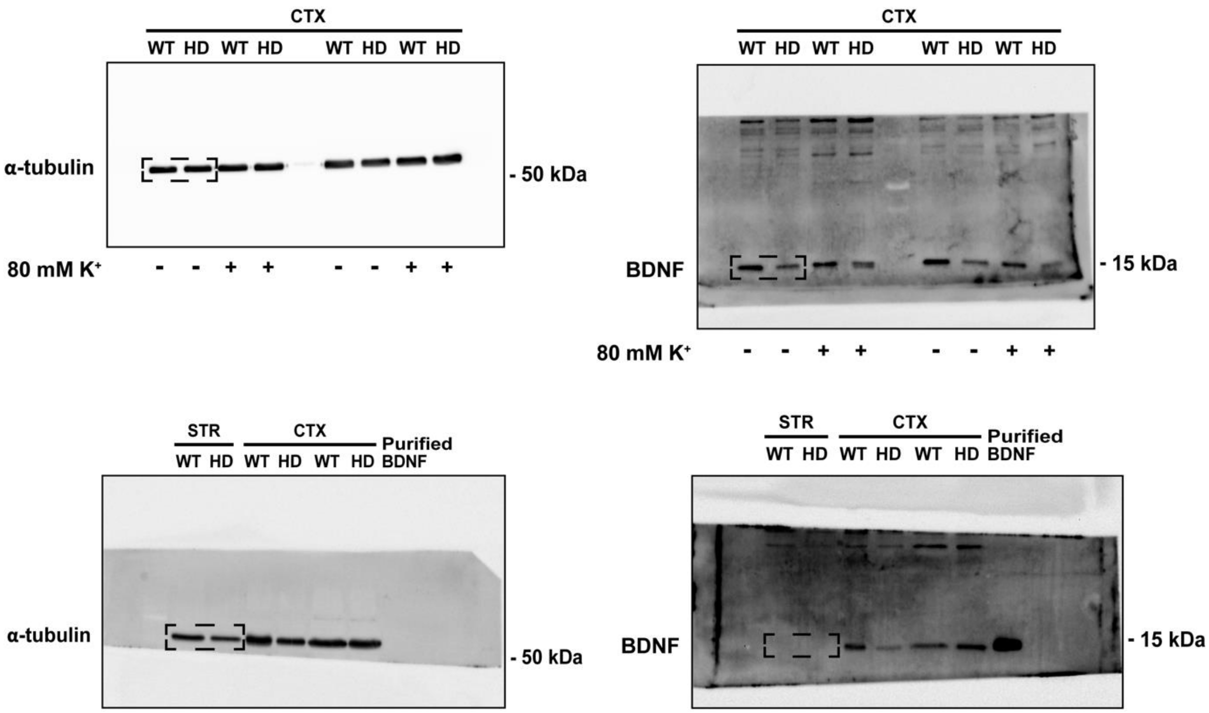

Supplementary Figure S6. Uncropped images presented with molecular weight ladders. Immunoblotting was performed on lysates from pure primary striatal (STR) and cortical (CTX) neurons at DIV14 using anti-BDNF antibody as shown in Figure 5a. Cropped areas are shown in dashed box region. Purified BDNF standard was diluted and loaded at 50ng/µl concentration.
